# CD36 – a plausible modifier of disease phenotype in familial adenomatous polyposis

**DOI:** 10.1186/s13053-018-0096-y

**Published:** 2018-07-28

**Authors:** Merran Holmes, Toni Connor, Christopher Oldmeadow, Peter G. Pockney, Rodney J. Scott, Bente A. Talseth-Palmer

**Affiliations:** 1 School of Medicine and Public Health, Faculty of Health and Medicine, University of Newcastle, Newcastle, NSW Australia; 2 Department of Surgery, John Hunter Hospital, Newcastle, Australia; 3 Pathology North, NSW Health Pathology, Newcastle, Australia; 4 Centre for Clinical Epidemiology and Biostatistics, University of Newcastle, Newcastle, NSW Australia; 5 School of Biomedical Science and Pharmacy, Faculty of Health and Medicine, University of Newcastle and Hunter Medical Research Institute, Newcastle, Australia; 6 Department of Clinical and Molecular Medicine, Faculty of Medicine and Health Sciences, Norwegian University of Science and Technology, Trondheim, Norway; 7 Clinic for Medicine, Møre og Romsdal Hospital Trust, Molde, Norway

**Keywords:** FAP, Disease phenotype, Polyposis, Modifier gene, CD36

## Abstract

**Background:**

Familial adenomatous polyposis (FAP) is a well characterised genetic predisposition to early onset colorectal cancer (CRC) that is characterised by polyposis of the colon and rectum. Animal models have consistently suggested the role of modifier genes in determining disease phenotype, yet none have been substantiated in the human population. The mouse homologue of *cluster of differentiation 36* (*CD36)* has been proposed as a modifier of disease in the MIN mouse model of FAP.

**Methods:**

Three single nucleotide polymorphisms (SNPs); rs1049673, rs1761667 and rs1984112 in *CD36,* have been investigated in 275 FAP patients to determine if they were associated with age of polyposis or risk of developing disease.

**Results:**

The results revealed a substantially lower age of polyposis diagnosis for patients belonging to the severe FAP group (harbouring *adenomatous polyposis coli (APC)* variants in the mutation cluster region (MCR)) and high age for patients in the attenuated familial adenomatous polyposis (AFAP) group for SNPs rs1761667 and rs1984112.

**Conclusions:**

This study provides evidence for patients belonging to the MCR and AFAP groups harbouring specific genotypes for SNPs in *CD36* to initiate screening/treatment for FAP at much earlier (MCR) and much later (AFAP) ages than the norm in today’s clinical practice. The findings need to be verified in an independent FAP patient cohort.

## Background

Familial adenomatous polyposis (FAP) is a rare autosomal dominantly inherited cancer syndrome that predisposes to colorectal cancer at unusually young ages, with disease penetrance that approaches 100% [1]. The diagnosis of FAP is based on the presence of hundreds to thousands of colorectal adenomatous polyps that appear in the colon and rectum during the first and second decades of life [2]. The disease is typically characterised by a carpeting of polyps within the colon and rectum and without appropriate prophylactic treatment approximately 95% of patients will develop CRC by the age of 40 years [2, 3].

FAP has been shown to be a result of germline mutations occurring in the tumour suppressor gene *adenomatous polyposis coli* (*APC*), which is located on chromosome 5q21–22.2 and is a key regulator of the Wnt signalling pathway [4]. Genotype/phenotype correlations are well described in FAP and are associated with variants that inactivate *APC* [5]. Genotype/phenotype studies reveal essentially two subgroups of FAP, one that predisposes to a milder form of disease known as attenuated FAP (AFAP) and the other more common form of the disease that is associated with the more severe phenotype [6, 7]. Pathogenic variants tend to occur within a mutation cluster region (MCR), bordered by codons 1286 and 1513, encompassing one specific variant (codon 1309) that is associated with the most severe phenotype [8]. Many reports have confirmed the severity of codon 1309 variants but not all patients with this mutation present with similar disease characteristics, with some patients displaying extremely severe polyposis whereas others have significantly less dense polyposis. These evidence suggests that there are other factors (genetic and/or environmental) influencing disease expression [5, 9].

Several studies have focused on the identification of candidate genes in mouse models of FAP (known as MOM - modifiers of MIN), reviewed in and recently 7 potential modifier genes (*CD36* being one of them) have been identified that affect tumour multiplicity in this model [10]. The mouse homologue of *CD36* has been proposed as a modifier of disease in the MIN mouse model of FAP [10]. *CD36,* a class B scavenger, located on chromosome 7q11.2, encodes a membrane glycoprotein found on epithelial cells of the breast, kidney and gut [11, 12] and has been shown to be involved in angiogenesis, atherosclerosis, inflammation, lipid metabolism and phagocytic clearance of apoptotic cells. There are several reports suggesting that *CD36* may play a role in affecting different disease phenotypes that include Alzheimer’s disease, heart disease, cancer, obesity, type 2 diabetes mellitus (T2DM) and associated metabolic disease [13–15].

Due to the modifier role of *CD36* in the mouse and its association with malignancy the aim of the current study was to determine whether three single nucleotide polymorphisms (SNPs) occurring in *CD36* were associated with modifying disease severity in a large cohort of molecularly diagnosed FAP patients*.*

## Methods

The patient cohort consisted of 275 individuals who carry a pathogenic germline mutation in *APC* (population I), belonging to 137 families. The patients were ascertained between 1997 and 2016, and analysed at Pathology North, Newcastle, NSW, Australia. From the 275 patients, 61 were diagnosed with AFAP due to their causative variant location, 34 had mutations in the mutation cluster region (MCR) and the remainder 179 was recognised as classic FAP. Mutation groups were divided according to polyposis phenotype; severe (APC MCR = codons 1250–1513), attenuated (APC AFAP = 5’end spanning exon 3 to 5, 3’distal end and those in exon 9) and intermediate (APC = the rest of the gene)). Population II (*n* = 214) excluded AFAP (attenuated) patients to see whether that influenced the results for the SNPs.

Several patients remained asymptomatic at the time of referral as they were too young to present with disease. The clinical data collected for this study included age of diagnosis of polyposis and age at last follow-up for patients undiagnosed with polyposis.

The 275 FAP patient samples were used for genotyping of SNPs in *CD36*; rs1049673 (C > G), rs1761667 (G > A) and rs1984112 (A > G) on chromosome 7q21.11 using TaqMan SNP assays (Applied Biosystems). SNP rs1049673 is located in exon 15 (3’-UTR), while the two others are intronic variants flanking exon 1A [16]. SNP rs1761667 has been shown to reduce protein expression, while 3’-UTR variants often contain regulatory regions that post-translationally influence gene expression [17]. The 3 SNPs are in strong LD with three haplotype blocks described by the HapMap database [18]. Thermo-cycling was undertaken according to the TaqMan SNP Genotyping Assay Protocol, involving; 10 min at 95 degrees; 40 cycles of 15 s at 95 degrees; and 1 min at 60 degrees. Results were read using the 7500 standard real-time PCR system (Applied Biosystems). Raw data was analysed using TaqMan Genotyper Software (Life Sciences, Foster City, CA).

A Pearson’s Chi-square test was used to evaluate deviation from the expected Hardy-Weinberg equilibrium (HWE). Statistical analysis was performed using Stata 12.1 (StataCorp LP, TX USA). We applied Bonferroni correction for multiple testing, resulting in a corrected significance threshold of *p* = 0.0167 (0.05 divided by the 3 SNPs tested). Due to the nature of the disease and prophylactic surgery, diagnosis of polyposis was used as endpoint for analysis. Variation in age of polyposis diagnosis between each SNP; wildtype genotype (homozygous for wildtype allele), heterozygote and variant genotype (homozygous for variant allele) and mutation group (based on mutation location as described above); was examined using Kaplan-Meier plots. Individuals free from polyposis were censored at their age at last follow up. Wilcoxon’s (Breslow), Log-rank and Tarone-Ware tests were used to examine homogeneity of the Kaplan-Meier plots. The log-rank test is more sensitive to differences later in time due to equal weighting over the curve, where the Wilcoxon weights the early differences higher than the later differences using the number at risk in the weighting. The Tarone-ware test uses the square root of the number at risk in the weighting. All three tests were required to be significant for results to be considered reliable. Cox regression models were used to provide a formal Wald-test of interaction (global interaction test) between APC mutation groups and SNPs genotypes, taking into account family ID as a group variable.

## Results

Of the 275 patients belonging to 137 families, 28 patients had a diagnosis of CRC and 182 a diagnosis of polyposis (population I). Of these there were 152 females and 123 males. The average age of polyposis was 36 years and for CRC 45 years. Of the 275 patients, 61 were classified as AFAP according to *APC* mutation location and the cohort was also analysed without AFAP patients (population II).

Patient samples that failed genotyping (24 for rs1049673, 13 for rs1761667 and 28 for rs1984112) were excluded from further analysis for the SNP in question. None of the SNPs deviated from HWE.

**Fig. 1:**
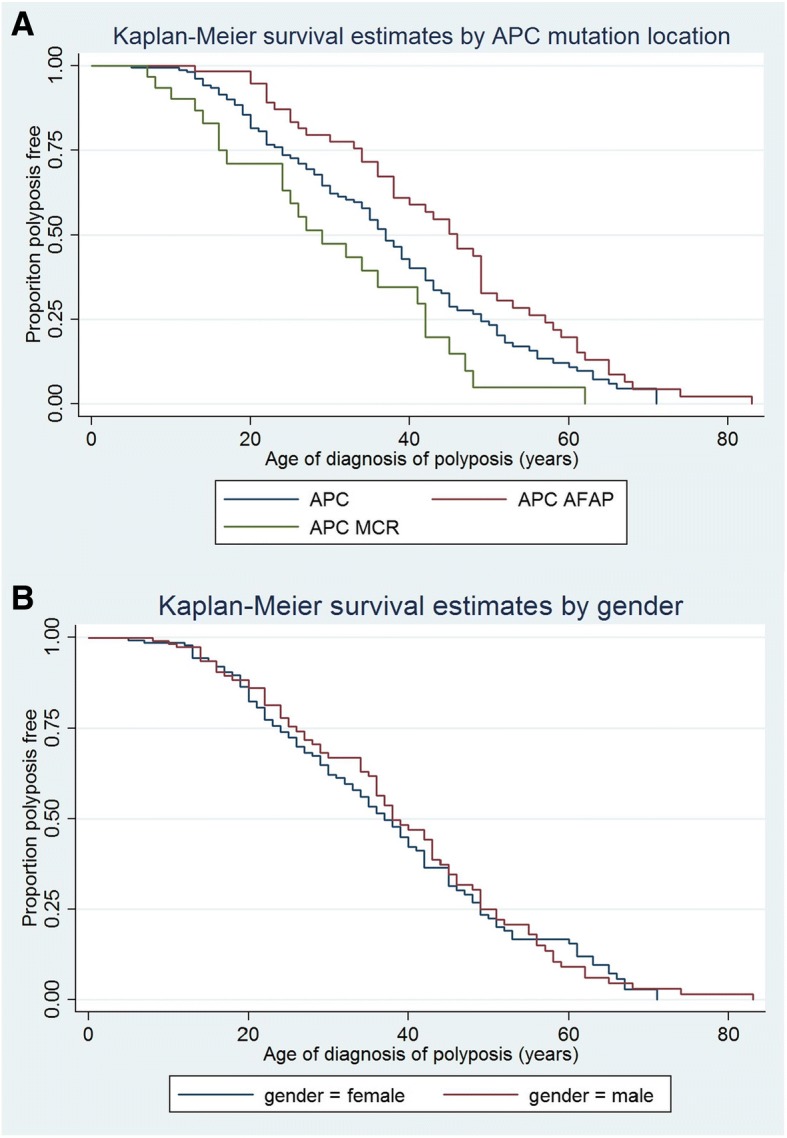
Shows the difference in age of diagnosis of polyposis between **a**) mutation-groups divided by APC mutation location (*n* = 275). APC = classic FAP, APC MCR = mutation cluster region (more severe phenotype) and APC AFAP (attenuated phenotype) – statistically significant; Log-rank *p* = 0.0010, Wilcoxon *p* = 0.0004 and Tarone ware *p* = 0.0005, and **b**) between female and males (n = 275). Not statistically different

**Fig. 2:**
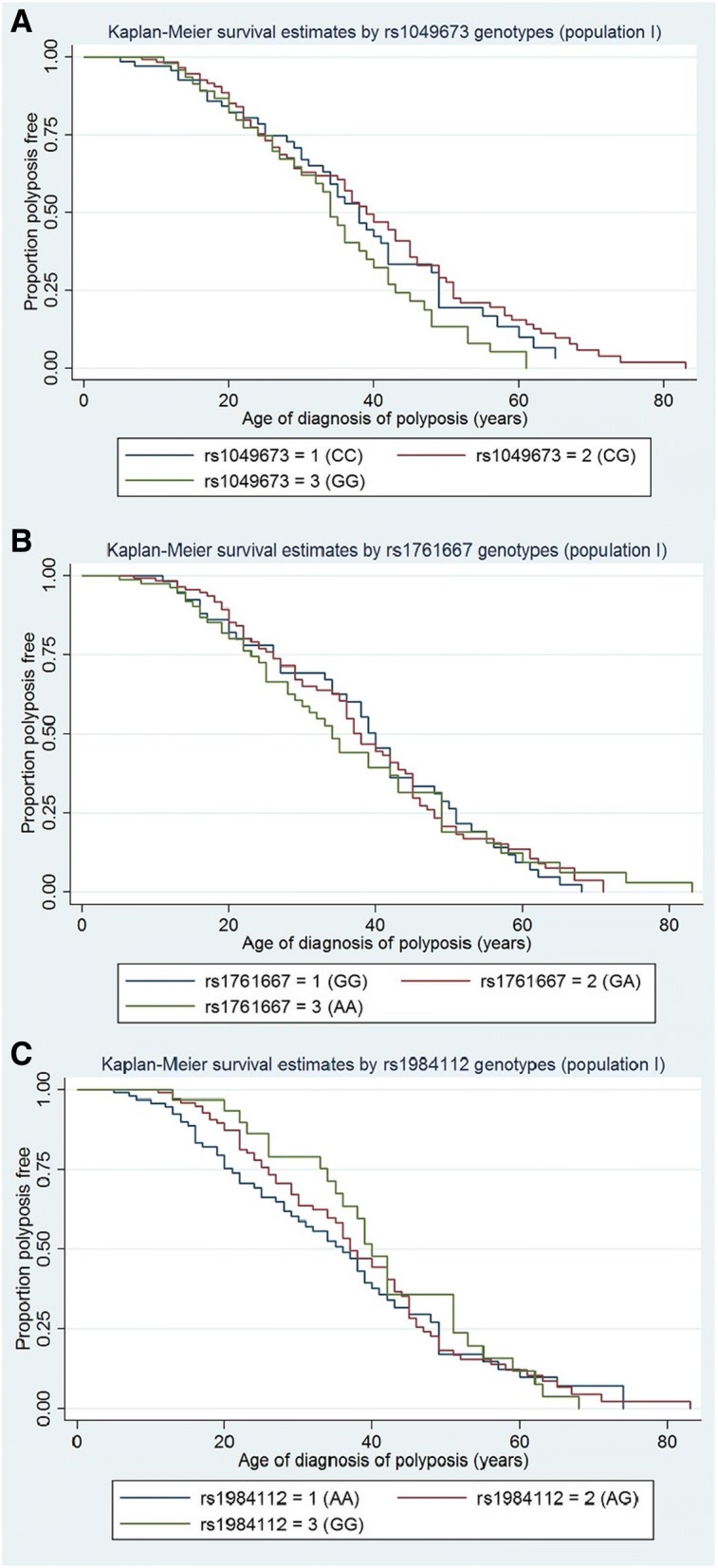
Kaplan-Meier curves for SNP **a**) rs1049676, **b**) rs1761667 and **c**) rs1984112 stratified by genotypes

In the sample cohort we observed a statistically significant difference between the phenotypic FAP groups (based on *APC* variant location) and age of polyposis diagnosis (log-rank *p* = 0.0010, Wilcoxon *p* = 0.0004 and Tarone ware *p* = 0.0005); age where 50% of population was polyposis free was 46 years for APC AFAP, 29 years for APC MCR and 37 years for APC, see Fig. 1. There was no statistically significant difference between males and females regarding age of diagnosis of polyposis in this cohort (see Fig. 2). Tests for equality of survival distributions between genotypes of the *CD36* SNPs rs1049673, rs1781667 and rs1984112 for population I was not statistically significant, shown in Table 1 and Fig. 2. Similar results were obtained for population II (data not shown).

**Table 1:** Population I (FAP and AFAP): The table lists genotypes for each SNP, number of polyposis cases and age where 50% of population I is polyposis free for each genotype

SNP	Genotypes^a^ (n) (polyposis cases (n)/age at 50%)				*p*-values		
	1	2	3	Total n (polyposis cases)	Log-Rank	Tarone-ware	Wilcoxon
rs1049673	74 (45/38)	124 (81/39)	53 (39/34)	251 (165)	0.0765	0.2210	0.4075
rs1761667	56 (44/40)	121 (82/38)	85 (46/34)	262 (172)	0.8891	0.5861	0.4527
rs1984112	102 (57/36)	108 (76/37)	37 (26/40)	247 (159)	0.6108	0.2215	0.0978

^a^Genotypes: 1 = wildtype, 2 = heterozygous and 3 = variant

To determine whether there was a differential effect of *CD36* genotypes depending on *APC* mutation location (mutation group) on age of diagnosis of polyposis we stratified the Kaplan-Meier curves into the three possible phenotypic groups (APC, APC MCR and APC AFAP), see Table 2. For two of the SNPs, rs1761667 and rs1984112, a trend (approaching statistical significance) shows that individuals harbouring the homozygous variant genotype and homozygous wildtype genotype have a remarkably lower age of polyposis diagnosis in patients with causative variants residing in the MCR (APC MCR), even though not statistically significant (see global interaction test in Table 2). Patients belonging to the APC MCR group, with the variant genotype (AA) for SNP rs1761667 and wildtype genotype (GG) for SNP rs1984112 (independent of each other) developed polyposis on average at 16 years of age, which is 13 years younger than the average age for this mutations group in the current sample cohort. Equally interesting is the high age of polyposis diagnosis (58 years) in patients belonging to the APC AFAP group harbouring the wildtype genotype (GG) for SNP rs1761667.

**Table 2:** Population I (FAP and AFAP): The table lists combination of genotypes (1 = wildtype genotype, 2 = heterozygote genotype and 3 = variant genotype) and phenotypic FAP group (1 = APC, 2 = APC MCR and 3 = APC AFAP) for each SNP and age where 50% of population I is polyposis free for each genotype. The mutation group/SNP interaction is the *p*-value from a joint test of the interaction parameters of a Cox model

SNP genotype	Age where 50% of population is polyposis free for each genotype^a^									Test equality of survivor functions			Cox model Mutationgroup/SNP interaction
	APC (mutation group 1)			APC MCR (mutation group 2)			APC AFAP (mutation group 3)			*p*-values			*p*-values Wald-test
	1	2	3	1	2	3	1	2	3	Log-Rank	Tarone-ware	Wilcoxon	
rs1049673	38	37	29	29	25	36	49	46	38	0.0082	0.0062	0.0057	0.3090
rs1761667	39	37	34	36	41	16	58	38	49	0.0010	0.0011	0.0015	0.0663
rs1984112	35	37	39	16	32	42	49	42	51	0.0036	0.0007	0.0001	0.4608

^a^Genotypes: 1 = wildtype, 2 = heterozygous and 3 = variant

## Discussion

The disease spectrum observed in patients with germline causative variants in *APC* has been associated with a genotype/phenotype correlation where some variants are linked to severe and others milder forms of the disease [9]. Notwithstanding, phenotype differences in patients with the same mutation are still observed and modifiers of disease severity still await discovery. This study was undertaken to determine whether *CD36* variants could account for phenotypic variation observed in FAP patients, and we have observed a remarkable low age of polyposis diagnosis (16 years) in patients with *APC* mutations in the MCR harbouring the variant (AA) genotype of rs1761667 or the wildtype (AA) genotype of rs1984112, independent of each other and a much higher age of polyposis diagnosis for patients harbouring the wildtype genotype (GG) of rs1761667 in the APC AFAP mutation group. This is 13 year younger and 12 years older than the average age of polyposis in this sample cohort for patients with mutations in the MCR and AFAP region, which were 29 and 46 years of age respectively, before taking into account the CD36 SNP genotypes.

FAP has long been categorised as a condition that affects younger people from their early teenage years. Virtually all patients will have developed polyposis a decade after the appearance of their first adenomas and, if left untreated, CRC by 40 years of age [1, 19]. But the phenomenon of anticipation has been described and age of disease onset decreases in successive generations [20]. Similar to the published penetrance estimates [6–8], the average age of polyposis and CRC diagnosis in our FAP population were 36 and 45 years of age, respectively, indicating that our sample cohort is consistent with other FAP populations. Our sample cohort clearly reveals a significant difference in disease expression among the three different phenotypic FAP groups; severe (APC MCR), intermediate (APC) and attenuated (APC AFAP) FAP, indicating that those individuals harbouring *APC* mutations in the MCR, should initiate screening/treatment at much earlier ages than other FAP or AFAP patients. But on top of this, the evidence presented herein suggest that patients belonging to the severe FAP group (APC MCR) who harbour the variant genotype for rs1761667 or wildtype genotype for rs1984112, should commence screening for polyposis at younger ages than their counterparts who do not carry these modifying alleles. Also interesting from a surgical perspective is that patients in the lower risk groups; especially AFAP patients harbouring the wildtype genotype for rs1761667 could be screened for longer before requiring prophylactic surgery.

*CD36* has been identified as a potentially interesting *Mom5* candidate [10] and the only gene found to be differentially expressed in the intestine in the mice used to reveal this modifier (B6 and 12P2 strains). CD36 encodes a scavenger receptor involved in fatty acid processing in the intestine [21]. *CD36* has also been associated with increased pro-inflammatory signalling and the production of reactive oxygen species [22], both known to influence tumour development. Previously, decreased expression of *CD36* has been observed in breast cancer and it has also been associated with worse prognosis in CRC patients [23, 24], lending weight to its role in tumour development.

For SNP rs1761667 the presence of the A (minor/variant) allele has been associated with decreased fat/sugar intake in obese children and increased BMI in T2DM [25]. Intriguingly, the AA genotype has also been associated with lower BMI [26] and high threshold of gustatory fat detection in obese women [27]. Overall, obesity has been unequivocally associated with an increased risk of colorectal cancer outside of the context of FAP. We were unable to obtain BMI information about our cohort of FAP patients at the time of polyposis development and are therefore unable to correlate these findings with an earlier age of disease onset.

Some limitations of our study should be considered. Due to the standard procedure of colectomy it is almost impossible to obtain accurate information about the age of cancer diagnosis in this population and as such we are unable to determine whether *CD36* variants are associated with cancer risk rather than the appearance of polyposis. The number of patients in this study remains limited and to prove beyond doubt the relationship between CD36 and disease development a larger number of FAP samples need to be tested. If these results can be verified in an independent cohort of FAP patients additional clinical information will be required to determine the precise role of *CD36* in ameliorating or promoting polyposis development. At present it remains to be revealed as to how *CD36* could influence adenoma development, one possible mechanism is its role as an endogenous inhibitor of angiogenesis. Lower levels of *CD36* predispose to higher levels of angiogenesis which is required for tumour growth^23^, which potentially explains the earlier age of disease onset in patients with variants that reduce promoter activity (i.e the rare allele of rs1761667 and common allele associated with rs1984112).

## Conclusions

If the findings from this study can be independently verified they will have important consequences with respect to the timing of prophylactic surgery for FAP patients and the overall quality of life for patients who have to undergo such a major life-changing event.

## Abbreviations

AFAP: attenuated familial adenomatous polyposis
APC: adenomatous polyposis coli
CD36: cluster of differentiation 36
CRC: colorectal cancer
FAP: familial adenomatous polyposis
HWE: Hardy Weinberg equilibrium
MOM: modifiers of MIM
MRC: mutation cluster region
SNPs: single nucleotide polymorphisms
T2DM: type 2 diabetes mellitus
